# Real-World Outcomes of Axicabtagene Ciloleucel for Treatment of Relapsed or Refractory Large B-Cell Lymphoma in Canada

**DOI:** 10.3390/curroncol33020085

**Published:** 2026-01-31

**Authors:** Christopher Lemieux, John Kuruvilla, Mona Shafey, Kelly Davison, Kristjan Paulson, Sue Z. L. Li, Lieven Billen, Francis Nissen, Hai-Lin Wang, Jenny J. Kim, Grace Lee, Zhen-Huan Hu, Brent Logan, Zhongyu Feng, Marcelo C. Pasquini, Kevin Hay

**Affiliations:** 1 CHU de Québec—Université Laval, Université Laval, Québec, QC G1R 2J6, Canada; 2 Princess Margaret Cancer Center, Toronto, ON M5S 1A8, Canada; 3 Department of Medicine, Arthur J.E. Child Comprehensive Cancer Centre, Calgary, AB T2N 5G2, Canada; 4 Royal Victoria Hospital, McGill University Health Centre, Montreal, QC H4A 3J1, Canada; 5 Section of Hematology/Oncology, Department of Internal Medicine, University of Manitoba, Winnipeg, MB R3T 2N2, Canada; 6 Kite, A Gilead Company, Santa Monica, CA 90404, USA; 7 Department of Medicine, CIBMTR (Center for International Blood and Marrow Transplant Research), Medical College of Wisconsin, Milwaukee, WI 53226, USA

**Keywords:** axicabtagene ciloleucel, axi-cel, CAR T, large B-cell lymphoma, LBCL, real-world registry

## Abstract

CD19 CAR T-cell therapy has significantly improved the survival of patients with relapsed or refractory large B cell lymphoma (R/R LBCL) and is considered standard of care for eligible patients in Canada. Axicabtagene ciloleucel (axi-cel) is an autologous CAR T-cell therapy, initially approved by Health Canada for adults with R/R LBCL after 2 or more lines of therapy. This multi-centre analysis, with registry data collected from CIBMTR, aims to present a Canadian perspective on the real-world experience of axi-cel in patients with R/R LBCL. With a median follow-up of 12.4 months, the best objective response rate (ORR) and complete response (CR) rate among all patients were 77% and 59%, respectively. At 12 months, estimated progression-free survival (PFS) and overall survival (OS) were 49% and 59%, respectively. Notably, the incidence and severity of adverse events were lower in this cohort compared to ZUMA-1 and other real-world reports, with CRS occurring in 77% (grade ≥ 3, 3%) and ICANS occurring in 38% (grade ≥ 3, 10%) of patients. Outcomes remained largely consistent across patient and disease characteristics. These findings demonstrate effectiveness and safety profiles comparable to international real-world studies and the ZUMA-1 trial, supporting the use of axi-cel as an effective treatment across broad Canadian populations.

## Simple Summary

Axicabtagene ciloleucel (axi-cel) is a type of chimeric antigen receptor T (CAR T)-cell therapy approved in Canada to treat adults with relapsed or refractory large B cell lymphoma, which is usually associated with a poor prognosis. In this retrospective study, we analyzed data from a large national registry database to evaluate the real-world effectiveness and safety outcomes of axi-cel across multiple Canadian centres. Our findings demonstrated that the effectiveness of axi-cel was comparable to those reported in clinical trial and real-world studies, with lower rates of adverse events. These findings support the ongoing use of axi-cel for the treatment of R/R LBCL in Canada.

## 1. Introduction

The advent of chimeric antigen receptor (CAR) T-cell therapy, a revolutionary cancer immunotherapy, has offered transformative outcomes for patients with a variety of hematological malignancies, including those with relapsed/refractory large B-cell lymphoma (R/R LBCL). These patients have typically had a very poor prognosis with a median overall survival (OS) of 6.3 months and an objective response rate (ORR) of 26% despite salvage chemotherapy and other interventions [1].

Axicabtagene ciloleucel (axi-cel) is an autologous anti-CD19 CAR T-cell therapy, which was approved by Health Canada in February 2019 for the treatment of adult patients with R/R LBCL after two or more lines of systemic therapy, including DLBCL not otherwise specified, primary mediastinal large B-cell lymphoma (PMBCL), high-grade B-cell lymphoma (HGBL), and DLBCL arising from follicular lymphoma [2]. The approval was based on the pivotal ZUMA-1 trial, which demonstrated that axi-cel offers a high ORR at 83% and complete response (CR) rate of 58% in adults with refractory LBCL [3]. In a 5-year follow-up analysis of the trial, median OS was reported at 25.8 months, with an estimated disease-specific survival of 51.0% at 5 years, demonstrating its curative potential [4]. Furthermore, in March 2023, the findings of the ZUMA-7 trial resulted in the approval of axi-cel in the second-line setting, specifically in patients with DLBCL or HGBL that is refractory to or that relapses within 12 months of first-line chemoimmunotherapy [2,5].

While the clinical trial data demonstrate impressive effectiveness outcomes, real-world evidence is needed to validate the effectiveness and safety of CAR T-cell therapy in broader patient populations, where eligibility criteria and monitoring are less controlled. Multiple real-world studies evaluated the use of axi-cel in patients with R/R LBCL [6,7,8,9,10,11]. Several US real-world studies have reported comparable effectiveness and safety outcomes of axi-cel to pivotal trials, despite large proportions of patients (43–57%) who would have been ineligible for ZUMA-1 [6,7]. Similarly, a national real-world study from the UK also demonstrated consistent effectiveness and a reduced trend of adverse events (AEs) over time, due to improved patient monitoring and better supportive care [9]. The GLA/DRST real-world data also further confirmed the real-world effectiveness and safety of axi-cel in a German R/R LBCL cohort [11].

In Canada, several single-centre studies have described their initial experience with toxicity and effectiveness outcomes of axi-cel comparable to the pivotal trials and international real-world data [12,13,14,15]. These studies highlight that, despite the complexity in administrating CAR T-cell therapy, and large referral catchment area or geography, patients can be effectively treated across Canada. These publications also highlight some of the challenges in access to CAR T-cell therapy, including radiation bridging therapy as a modality to achieve disease control and impact of travel distance on timely CAR T cell infusion [12,15,16]. In contrast to the US and Europe, Canada currently lacks studies to assess outcomes across the broader Canadian population.

Here, we aim to characterize the real-world effectiveness and safety of axi-cel in patients with R/R LBCL treated across Canadian CAR T centres in third- and later-line settings and provide a national perspective on the Canadian experience.

## 2. Materials and Methods

### 2.1. Study Design and Patient Selection

This registry-based, noninterventional cohort study is a secondary use of real-world data from the observational database of the Center for International Blood and Marrow Transplant Research (CIBMTR^®^, Milwaukee, WI, USA) registry in collaboration with Cell Therapy Transplant Canada (CTTC) for adult patients (≥18 years) receiving commercial axi-cel for R/R LBCL between February 2020 and June 2023 in Canada (data cut-off 1 October 2024). Patients who died or discontinued prior to data cut-off were also included. All patients provided informed consent for participation in the CIBMTR for research studies, and the use of data for research was approved and overseen by the National Marrow Donor Program (NMDP) central institutional review board.

Information on patient, treatment and disease characteristics were reported by participating treatment centres at the time of axi-cel infusion, along with follow-up forms for 100-day, 6 months and then annually following infusion. Eligible patients must also have had both pre-infusion and at least 100-day follow-up data available. Data reporting requirements are further described in Supplemental Materials.

### 2.2. Treatment and Endpoint Assessments

Effectiveness endpoints assessed included ORR, CR rate, partial response (PR) rate, time to overall response (TTOR), time to complete response (TTCR), duration of response (DOR), duration of complete response (DOCR), progression-free survival (PFS), OS, and relapse or progressive disease (REL/PD). Disease response was assessed according to the Lugano classification scheme [5]. Relapse and disease progression were determined by the investigator at the treatment centre.

These endpoints were defined as follows: TTOR—Time from date of infusion to the initial response for subjects with PR or CR as best response. Post-infusion REL/PD or death were treated as competing risk events. TTCR—Time from date of infusion to initial response of CR, for subjects with CR as best response. DOR—Time from first CR or PR to REL/PD through radiological and/or clinical assessment, or death due to any causes (i.e., PFS since initial response as CR/PR) (only applies to subjects with CR (including CCR) or PR as best response). DOCR—Time from first CR to REL/PD through radiological and/or clinical assessment, or death due to any causes (i.e., PFS since initial response as CR) (only applies to subjects with CR (including CCR) as best response). PFS—Time from the first axi-cel infusion to the earliest documented REL/RD through imaging assessment (CT, PET, MRI) and/or clinical/hematologic assessment (including pathology and laboratory assessment, as well as physical examination) or death due to any cause. OS—Time from the first axi-cel infusion to death due to any cause. REL/PD—Time from the first axi-cel infusion to earliest documented REL/PD through imaging assessment (CT, PET, MRI) and/or clinical/hematologic assessment (including pathology and laboratory assessment, as well as physical examination).

Safety outcomes assessed were CRS and ICANS as per grading by the American Society for Transplantation and Cellular Therapy (ASTCT) consensus criteria [17], prolonged cytopenia (those failed to resolve within the first 30 days after infusion), clinically significant infections (diagnosed after the initial infusion of axi-cel that requires treatment), and non-relapse mortality (NRM).

### 2.3. Statistical Analysis

Dichotomous outcomes (ORR, CR rate, PR rate, CRS, ICANS, prolonged cytopenia, clinically relevant infections, and time from infusion to infection) were summarized using percentages with 95% Clopper–Pearson confidence intervals. Time to event outcomes without competing risk (DOR, DOCR, PFS, OS) were summarized using the Kaplan–Meier estimator. Time to event outcomes with competing risk (TTOR, TTCR, REL/PD, NRM) were summarized using the cumulative incidence function. Reported *p*-values were two-sided, with a *p*-value of <0.05 considered statistically significant. All statistical analyses were performed using SAS^®^ software, version 9.4 M8 (SAS Institute Inc., Cary, NC, USA).

## 3. Results

### 3.1. Patients

**Figure A1:**
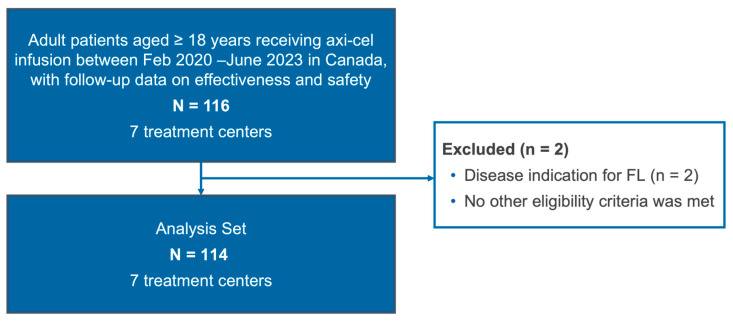
Patient disposition diagram. Abbreviations: Axi-cel, axicabtagene ciloleucel; FL, follicular lymphoma.

A total of 114 eligible patients were identified in the CIBMTR registry (Appendix A, Figure A1). At the time of data cut-off (1 October 2024), the median follow-up was 12.4 months (95% confidence interval [CI], 12.1–13.0). Patients were treated across 7 CAR T treatment centres. The median age of the patients was 63 years (range 20–81; 39% ≥ 65 years and 11% ≥ 75 years), and 67% were male (Table 1). The majority of the patients (94%) demonstrated a baseline ECOG performance score of 0 or 1, and clinically significant co-morbidities were present in 64% of patients (Table 1). Approximately, one-third (34%) of the patients would have been ineligible for ZUMA-1, mainly due to organ impairment (Appendix B, Table A1).

At initial diagnosis, histology subtypes included 92 diffuse LBCL (81%), 17 high-grade BCL (15%), 4 primary mediastinal BCL (4%), and 1 monomorphic PTLD (<1%). The majority of patients (77%) were diagnosed with stage III or IV disease, and 81% of the patients had extranodal involvement. Prior to infusion, approximately half of the patients (54%) demonstrated elevated lactate dehydrogenase (LDH) levels. Among patients with reported parameters to calculate CAR-HEMATOTOX score (*n* = 61), the majority (70%) of the patients had a score of 2+, indicating higher risk of CAR T-cell-related hematotoxicity [19]. Most (98%) of the patients presented non-bulky disease, with extranodal involvement being observed in 56% of the patients. Patients had received a median of 3 prior lines of therapy (IQR, 2–4), and 30% received autologous HCT. In addition, 40% of patients were treated with axi-cel within 12 months of initial disease diagnosis. Prior to leukapheresis, 81% of the patients were refractory after last line of therapy. Median time from leukapheresis to infusion was 32 days (IQR, 28–34), during which 51 patients (52%) received bridging therapy (35% systemic, 34% radiation). Among patients who received bridging therapy with reported response data, 9% and 41% achieved a CR and PR, respectively.

### 3.2. Effectiveness

**Figure 1:**
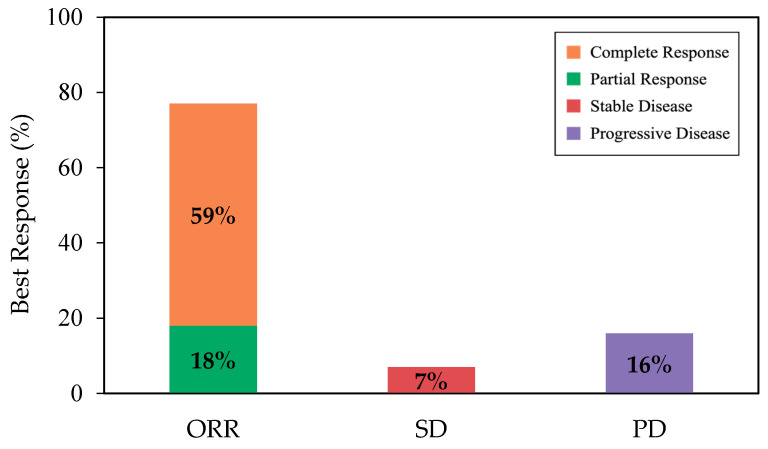
The rate of best ORR (as complete response + partial response), SD, and PD, among all patients. Abbreviations: ORR, overall response rate; PD, progressive disease; SD, stable disease.

**Figure 2:**
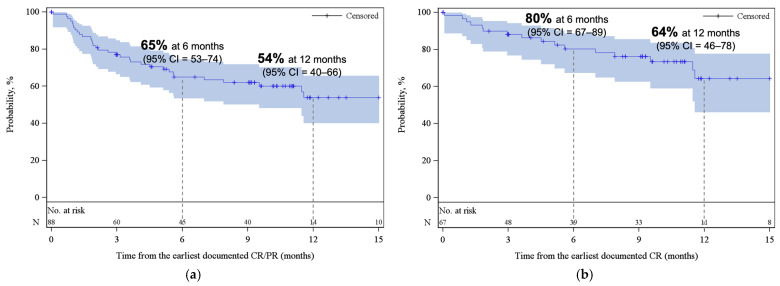
Kaplan–Meier estimates for duration of response (**a**) among patients who achieved CR/PR as best response and (**b**) among patients who achieved CR as best response. The shaded areas represent the 95% CI. The dotted lines represent the estimated DOR at 6 and 12 months. Abbreviations: CI, confidence interval; CR, complete response; PR, partial response.

**Figure A2:**
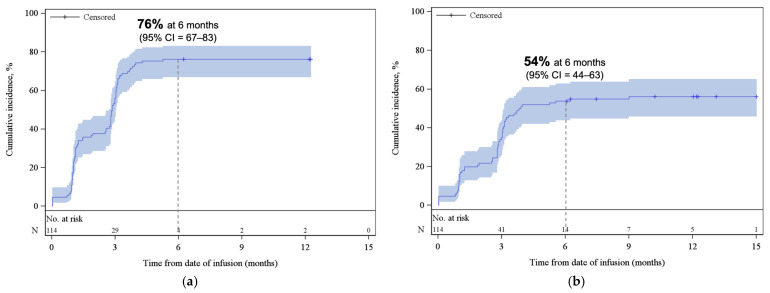
Cumulative incidence function plot for (**a**) ORR and (**b**) CR. REL/PD or death were treated as competing risks. The shaded areas represent the 95% CI. The dotted lines represent the estimated cumulative incidence of (**a**) ORR and (**b**) CR at 6 months. Abbreviations: CI, confidence interval; CR, complete response; ORR, overall response rate.

With a median follow-up of 12.4 months from infusion, the best ORR and CR among all patients were 77% (95% CI, 68–85) and 59% (95% CI, 49–68), respectively (Figure 1). Cumulative incidences of ORR and CR at 6 months were 76% (95% CI, 67–83) and 54% (95% CI, 44–63), respectively (Appendix A, Figure A2). While the median DOR was not reached, among responders, 65% (95% CI, 53–74) and 54% (95% CI, 40–66) remained in response for 6 months and 12 months, respectively (Figure 2a). Among patients who achieved CR, 80% (95% CI, 67–89) and 64% (95% CI, 46–78) remained in response for ≥6 months and 12 months, respectively (Figure 2b).

**Figure 3:**
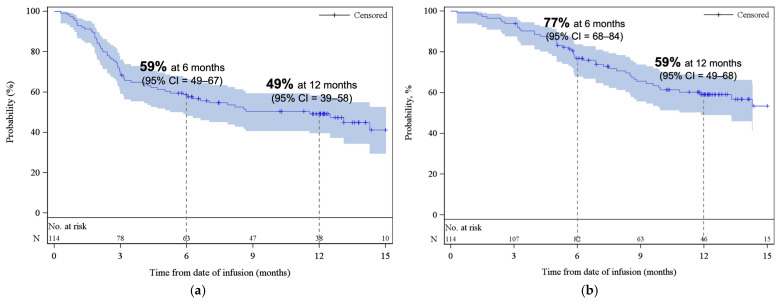
Kaplan–Meier estimates of (**a**) progression-free survival and (**b**) overall survival. The shaded areas represent the 95% CI. The dotted lines represent the estimated (**a**) PFS and (**b**) OS at 6 and 12 months. Abbreviations: CI, confidence interval.

**Figure A3:**
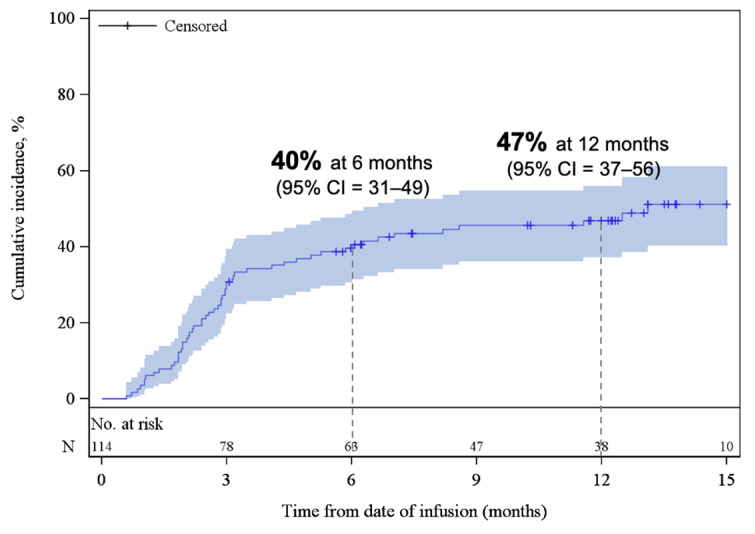
Cumulative incidence function plot for REL/PD. The shaded areas represent the 95% CI. The dotted lines represent the estimated cumulative incidence of REL/PD at 6 and 12 months. Abbreviations: CI, confidence interval; REL/PD, relapse or progressive disease.

The median PFS was 11.6 months (95% CI, 5.3–NE), with estimated PFS rates of 59% (95% CI, 49–67) at 6 months and 49% (95% CI, 39–58) at 12 months (Figure 3a). The median OS was 18.2 months (95% CI, 11.9–NE), with estimated OS rates of 77% (95% CI, 68–84) at 6 months and 59% (95% CI, 49–68) at 12 months (Figure 3b). At the time of data cut-off, 59% of patients remained alive. At 3 and 6 months, the cumulative incidence of REL/PD were 31% (95% CI, 22–39) and 40% (95% CI, 31–49), respectively (Appendix A, Figure A3). The response rates and survival rates were largely consistent across subgroups of key baseline and disease characteristics, including HCT-CI index, and age at infusion (Supplemental Table S1). Only ECOG PS of ≥2 appeared to be associated with inferior outcomes; however, this subgroup contained a small number of patients (*n* = 7).

### 3.3. Safety

Among patients with reported data, CRS occurred in 77% of patients (95% CI, 68–85), with grade ≥ 3 events in 3 patients (3%) and no reported cases of Grade 4 or higher CRS (Table 2). The median time from infusion to the onset of CRS was 4 days (IQR, 2–6), and median time from onset to resolution was 5 days (IQR, 4–8).

Neurotoxicity occurred in 38% of patients (95% CI, 29–48), with ICANS Grade ≥ 3 occurring in 10% patients and no reported cases of Grade 4 or higher ICANS. The median time from infusion to onset was 8 days (IQR, 6–8), and median time to resolution was 7 days (IQR, 4–9.5).

Of the 98 patients with complete data on both CRS and neurotoxicity, 72 patients (73%) were without CRS and ICANS at Day 14 and beyond, including patients whose toxicities resolved prior to Day 14 (48 patients, 49%), as well as patients without any onset of either CRS or neurotoxicity (24 patients, 24%). The remaining 26 patients either experienced ongoing CRS and/or neurotoxicities on Day 14 (23 patients; 23%) or had onset CRS or ICANS after Day 14 (3 patients; 3%). Among the three patients with CRS and/or ICANS onset after Day 14, two patients had CRS onset on Day 15, one with a maximum grade of 1 and the other a maximum grade of 2. One patient also experienced neurotoxicity onset on Day 15 with a maximum ICANS Grade of 3 and resolution of ICANS on Day 23. No specific patient or disease characteristic related to late-onset events was identified. Among patients experiencing CRS and/or ICANS (*n* = 87), 44% received corticosteroids, 85% received tocilizumab, and 5% received anakinra. Nearly all events associated with CRS were resolved, except for two cases ongoing at data cut-off. All ICANS events were fully resolved.

Prolonged cytopenia after Day 30 occurred in 17% of patients (12% neutropenia, 5% thrombocytopenia). Clinically significant infection that required treatment occurred in 46% of patients. Median time from infusion to infection was 0.9 months (25th to 75th percentile, 0.2–2.4). The occurrences of safety events remained largely consistent across different subgroups. NRM occurred in 2% (95% CI, <1–6) by month 6 and 4% (95% CI, 1–9) by month 12. Among all patients who received infusion, 47 patients (41%) died during follow-up; this was due to primary disease in 42 patients (89%), organ failure in 3 patients (6%; 2 pulmonary failures, 1 cardiac failure), and malignancy in 2 patients (4%) (Appendix B, Table A2). The safety outcomes remained largely consistent across various subgroups (Supplemental Table S2).

## 4. Discussion

This is the first multi-centre national registry study of patients on axi-cel for R/R LBCL in real-world settings in Canada. Our findings demonstrate an ORR of 77% and a CR rate of 59%, which are comparable to outcomes reported in the pivotal ZUMA-1 trial (ORR of 82%, CR rate of 54%) [3] and other real-world datasets, with CR rates ranging from 42 to 78% [6,9,11,12,13,14,15,20]. In particular, our findings strongly align with a Canadian single-centre study that reported a 12-month response rate of 72% with axi-cel [12]. In contrast, another Canadian single-centre study reported a poor early response rate (3-month ORR of 47% with axi-cel). This discrepancy may be attributed to the high prevalence of bulky disease in the patient cohort, which was identified as a strong predictor of poor early response [14]. The variability in efficacy across centres may be attributed to the small cohorts included in single-centre reports, which can increase variability, and heterogeneity in patient characteristics, particularly disease status at the time of infusion. Importantly, these outcomes were achieved despite 34% of the patients not meeting ZUMA-1 eligibility criteria, largely due to organ impairment. Additionally, 64% of patients presented clinically significant comorbidities, and 25% were 70 years or older, highlighting the robustness of axi-cel effectiveness in a broader patient population. Moreover, effectiveness outcomes were largely consistent across subgroups, including age and HCT-CI index at infusion, which further supports the finding that older patients and non-transplant-eligible patients can still benefit from axi-cel [21,22,23,24].

Overall, the incidence of CRS and ICANS observed in our study (77% and 38%, respectively) was largely consistent with those reported in the clinical trial and other real-world studies [3,6,7,9,10,11], although some variability was observed compared to other Canadian single-centre reports. In particular, the overall incidence of CRS and ICANS was lower than that reported in other Canadian single-centre studies [12,15,16]. The multi-centre nature of our study may potentially normalize centre-to-centre differences in patient selection and toxicity management, thereby providing a wider perspective of Canadian experiences with axi-cel. Importantly, the occurrences of grade ≥3 AEs (3% grade ≥3 CRS and 10% grade ≥3 ICANS) were markedly lower compared to the ZUMA-1 trial (11% grade ≥3 CRS and 32% grade ≥3 ICANS), which can be attributed to several factors. First, most institutions in the current study implemented an early toxicity management similar to the ZUMA-1 cohort 4, where patients received tocilizumab and/or corticosteroids earlier than the ZUMA-1 cohorts 1 and 2 [25]. This suggests that earlier toxicity intervention may reduce the severity and incidence of high-grade CRS/ICANS without compromising the effectiveness of axi-cel. In addition, unlike the ZUMA-1 trial, where bridging therapy was not permitted, approximately half (52%) of the patients in this study received bridging therapy, where systemic therapy and/or radiation were most frequently used. Among patients with reported data on response to bridging therapy, 50% achieved CR or PR as best response to bridging therapy. Previous studies suggest that response to bridging therapy is strongly correlated with more favourable CAR T outcomes [12,26,27], suggesting that disease control prior to axi-cel can improve the treatment-related toxicities seen in this study.

A small number of patients had onset of CRS or ICANS after Day 14 (3 out of 98 patients), which is consistent with recent real-world evidence looking at new-onset CRS/ICANS beyond Day 14 [28]. Furthermore, most patients (73%) did not experience CRS/ICANS symptoms at Day 14 and beyond. These data are notable given the Food and Drug Administration’s (FDA) decision to eliminate the Risk Evaluation and Mitigation Strategy (REMS) programme for autologous CAR T therapies [29]. The FDA no longer requires US CAR T treatment centres to be specially certified and has reduced the requirement for patients to remain within proximity of a healthcare facility from 4 weeks to 2 weeks. This may improve access to CAR T in the US and may be relevant to Canadian regulators as well.

Canada’s large geography and limited number of CAR T centres present various challenges to access, especially for patients treated outside of their province of residence or those from remote regions. In addition, shipping commercial CAR T products from the US to Canada may contribute to longer vein-to-vein time. While the median vein-to-time in our study was longer compared to the US CIMBTR report (32 days vs. 27 days), the majority of patients (87%) received infusion within <40 days after leukapheresis, a timeframe previously associated with favourable CAR T outcomes [5]. Interestingly, a study from the Ottawa CAR T centre reported that patients treated out-of-province experienced significantly longer times from relapse to referral compared to in-province patients (34 days vs. 9 days) [15]. However, the vein-to-vein time did not differ between the two groups, suggesting that inequality in timely access to CAR T in Canada may extend beyond vein-to-vein time. Multi-centre analyses are required to assess the impact of patient location on the timeline of axi-cel infusion and their outcomes.

There are several limitations to this study. Despite being a national registry, reporting to the CIBMTR/CTTC registry has not been nationally mandated; thus, not all authorized CAR T centres in Canada actively participated in reporting data. Specific CIBMTR reporting forms were required to be completed to ensure sufficient data capture for analysis. Despite this, data for various parameters remain incomplete, which may impact data interpretation. Increased resources and support are needed for data managers across Canadian sites to improve the completeness and accuracy of registry data. Another limitation within the CIBMTR datasets is that reoccurrences of safety events are not specifically captured. Whether CRS or ICANS recurs in the same patient after an initial resolution cannot be assessed. Whether the data reported here on the onset and resolution of these events represents the first instances only or includes recurrences in patients who experience them is also unclear. As such, the data on the number of patients who are without CRS or ICANS at Day 14 and beyond should be interpreted with caution. Recent data from several axi-cel clinical trials suggest that in patients who have durable remission of CRS/ICANS, defined as 3 consecutive days, the risk of recurrence is low [30].

This report focuses on third-line patients who were refractory to, or had relapsed after, two or more prior lines of systemic therapy. Prior to the approval of CAR T-cell therapy in second-line, patients who were refractory to or relapsed within 12 months of first-line therapy who responded to second line salvage chemotherapy would have proceeded to the standard second-line approach of high-dose therapy, followed by autologous SCT. Consequently, CAR T-cell therapy would have been administered only after a patient was unable to proceed to transplant (e.g., did not achieve a response to salvage or not eligible for transplant) or had relapsed following transplant. Therefore, no comparison between CAR T-cell therapy versus autologous SCT could be performed in this study. Notably, 30% of patients in this cohort had received a prior autologous transplant, as shown in Table 1.

The treatment landscape for R/R LBCL is rapidly evolving with the introduction of additional treatment options, such as bispecific antibodies (BsAbs) and CAR T treatment in earlier lines of therapy [31,32]. As of this report, second-line axi-cel is available in Canada. However, during the infusion period of data reporting, there was no access to axi-cel in the second-line setting. The ZUMA-7 trial demonstrated that axi-cel significantly improves EFS, response rates, and OS compared to standard care (chemoimmunotherapy followed by auto-SCT in those who are responsive), suggesting that earlier introduction of axi-cel may significantly improve outcomes [5]. While treatment sequencing remains an increasingly important question with the advent of additional therapeutic options for these patients, the use of BsAbs is currently recommended in those ineligible for, or relapsing after, CAR T therapy [31,32]. Future studies are needed to assess the real-world experience of axi-cel in earlier lines of therapy.

Overall, our findings demonstrate comparable effectiveness and safety profiles in a broad Canadian patient population consistent with those observed in trials and in international real-world studies, supporting the ongoing use of axi-cel for the treatment of R/R LBCL in Canada.

## Figures and Tables

**Table 1:** Baseline patient and disease characteristics.

Baseline Characteristics	Overall (*N* = 114)
Age at infusion, median (range), years	63 (20–81)
≥65 years	45 (39)
≥70 years	28 (25)
≥75 years	12 (11)
Sex, male	76 (67)
ECOG performance status 0 or 1 prior to CT	107 (94)
Clinically significant co-morbidity ^a^	
Yes	73 (64)
Arrythmia, any history	6 (5)
Cardiac, any history	14 (12)
Hepatic (moderate/severe), any history or at time of infusion	1 (<1)
Hepatic (mild), any history or at time of infusion	6 (5)
Pulmonary (severe), at time of infusion	4 (4)
Renal (moderate/severe), at time of infusion or prior renal transplant	2 (2)
Infection requiring antimicrobial treatment, continuation after Day 1	4 (4)
Diabetes requiring non-diet treatment, in the last 4 weeks	18 (16)
Severe underweight, with BMI < 20 kg/m^2^, at time of infusion	11 (10)
No	41 (36)
ZUMA-1 eligible	75 (66)
**Disease Characteristics at Initial Diagnosis, *n* (%)**	
Disease histology at initial diagnosis	
DLBCL	92 (81)
PMBCL	4 (4)
HGBL	17 (15)
Monomorphic PTLD	1 (<1)
Histologic transformation	32 (28)
Double/triple hit ^b^	15 (24)
Ann Arbor stage (stage III or IV) ^c^	69 (77)
Extranodal involvement ^d^	
Yes	66 (81)
2 or more extranodal sites	33 (41)
CNS	4 (6)
**Disease Characteristic Prior to Infusion, *n* (%)**	
Elevated LDH ^e^	58 (54)
C-reactive protein, mg/dL ^f^	
Mean (SD)	0.3 (1.96)
Median (range)	0.0 (0.0–15.9)
Serum ferritin, ng/mL ^g^	
Mean (SD)	520.6 (517.19)
Median (range)	383.5 (13.0–2949.0)
Hemoglobin, g/dL	
Mean (SD)	1.1 (0.20)
Median (range)	1.1 (0.5–1.5)
WBC, ×10^9^/L	
Mean (SD)	5.4 (2.94)
Median (range)	5.0 (0.6–17.1)
ANC, ×10^9^/L ^h^	
Mean (SD)	3.8 (2.57)
Median (range)	3.3 (0.1–14.0)
Platelet count, ×10^9^/L	
Mean (SD)	173.5 (95.77)
Median (range)	163.5 (15.0–636.0)
CAR-HEMATOTOX score ^i^	
Low (0–1)	18 (30)
High (2+)	43 (70)
Bulky disease ^j^	
Non-bulky (longest diameter of the largest nodal mass < 10 cm)	87 (98)
Bulky (longest diameter of the largest nodal mass ≥ 10 cm)	2 (2)
Extranodal involvement ^d^	
Yes	45 (56)
2 or more extranodal sites	22 (27)
CNS	3 (7)
**Treatment History**	
Prior lines of therapy, median (IQR) ^k^	3 (2–4)
Prior history of autologous HCT ^l^	34 (30)
Refractory after last line of therapy prior to leukapheresis ^m^	59 (81)
Time from initial diagnosis to infusion, median (IQR), months	15 (9–33)
<12 months	46 (40)
Time from leukapheresis to infusion, median (IQR), days ^n^	32 (28–34)
<40 days	97 (87)
Bridging therapy ^o^	
No bridging	47 (48)
Any	51 (52)
Systemic	34 (35)
Radiation	33 (34)
Best response to bridging therapy ^p^	
CR	3 (9)
PR	13 (41)
No response/stable disease	4 (13)
PD	12 (38)
Year of axi-cel infusion	
2020	1 (<1)
2021	12 (11)
2022	48 (42)
2023	53 (46)

^a^ According to Hematopoietic Cell Transplantation Comorbidity Index (HCT-CI) [18]. ^b^ Among patients with reported data (*n* = 62). ^c^ Among patients with reported data (*n* = 90). ^d^ Among patients with reported data (*n* = 81). ^e^ Among patients with reported data (*n* = 107). ^f^ Among patients with reported data (*n* = 66). ^g^ Among patients with reported data (*n* = 70). ^h^ Among patients with reported data (*n* = 109). ^i^ Among patients with reported data (*n* = 61). ^j^ Among patients with reported data (*n* = 89). ^k^ Among patients with reported data (*n* = 102). ^l^ Among patients with reported data (*n* = 113). ^m^ Among patients with reported data (*n* = 73). ^n^ Among patients with reported data (*n* = 111). ^o^ Among patients with reported data (*n* = 98). Systemic therapy and radiotherapy categories were not mutually exclusive. ^p^ Among patients with reported data (*n* = 95). Systemic therapy and radiotherapy categories were not mutually exclusive. Abbreviations: ANC, absolute neutrophil count; axi-cel, axicabtagene ciloleucel; BMI, body mass index; CNS, central nervous system; CR, complete response; CT, chemotherapy; DLBCL, diffuse large B-cell lymphoma; ECOG, Eastern Cooperative Oncology Group; HCT, hematopoietic cell transplantation; HGBL, high-grade B-cell lymphoma; IQR, interquartile range; LDH, lactate dehydrogenase; PD, progressive disease; PMBCL, primary mediastinal B-cell lymphoma; PR, partial response; PTLD, post-transplant lymphoproliferative disorder; SD, standard deviation; WBC, white blood cell.

**Table 2:** Safety outcomes.

CRS, *n* (%)	
Any grade CRS ^a^	81 (77)
Grade 1	46 (44)
Grade 2	32 (30)
Grade 3	3 (3)
Grade ≥ 4	0 (0)
Median time from infusion to CRS onset, days (IQR)	4 (2–6)
Median time from CRS onset to resolution, days (IQR)	5 (4–8)
Total CRS resolution rate	79 (98)
**Neurotoxicity, *n* (%)**	
Any grade ICANS ^b^	40 (38)
Grade 1	21 (20)
Grade 2	8 (8)
Grade 3	11 (10)
Grade ≥ 4	0 (0)
Median time from infusion to ICANS onset, days (IQR)	8 (6–8)
Median time from ICANS onset to resolution, days (IQR)	7 (4–9.5)
Total ICANS resolution rate	40 (100)
**AE Management of CRS or ICANS (%)** ^c^	
Therapies	
Corticosteroids	38 (44)
Tocilizumab	73 (84)
Anakinra	4 (5)
**Other Adverse Events of Interest, *n* (%)**	
Prolonged cytopenia ^d^	19 (17)
Neutropenia	13 (12)
Thrombocytopenia	6 (5)
Clinically significant infection	53 (46)
Type of 1st secondary malignancy	
No secondary malignancy	109 (96)
Treatment-related myeloid neoplasm	3 (3)
Acute myeloid leukemia (AML/ANLL)	1 (20)
Myelodysplasia (MDS)	2 (40)
Other	2 (2)
Gastrointestinal malignancy	1 (20)
Soft tissue	1 (20)
**Non-Relapse Mortality**	
Month 6	2 (<1–6)
Month 12	4 (1–9)

^a^ Among patients with reported data (*n* = 105). ^b^ Among patients with reported data (*n* = 106). ^c^ Among patients who experienced CRS and/or ICANS, (*n* = 87). ^d^ Among patients who survived Day 30 (*n* = 113). Abbreviations: AE, adverse event; AML, acute myeloid leukemia; ANLL, acute non-lymphocytic leukemia; CI, confidence interval; CRS, cytokine release syndrome; ICANS, immune effector cell-associated neurotoxicity syndrome; IQR, interquartile range; MDS, myelodysplasia.

## Data Availability

The data presented in this study are included in the article/Supplementary Materials. Further inquiries can be directed to the corresponding author.

## Supplementary Materials

The following supporting information can be downloaded at https://www.mdpi.com/article/10.3390/curroncol33020085/s1, Supplemental Methods; Table S1: Efficacy outcomes by subgroups; Table S2: Safety outcomes by subgroups.

## Abbreviations

The following abbreviations are used in this manuscript:

AE	Adverse events
ALK	Anaplastic lymphoma kinase
AML	Acute myeloid leukemia
ANLL	Acute non-lymphomic leukemia
ANC	Absolute neutrophil count
ASTCT	American Society for Transplantation and Cellular Therapy
axi-cel	Axicabtagene ciloleucel
BCL	B-cell lymphoma
BMI	Body mass index
BsAbs	Bispecific antibodies
CAR	Chimeric antigen receptor
CI	Confidence Interval
CIBMTR	Center for International Blood and Marrow Transplant Research
CNS	Central nervous system
CR	Complete response
CRS	Cytokine release syndrome
CT	Chemotherapy
CTTC	Cell Therapy Transplant Canada
DLBCL	Diffuse large B-cell lymphoma
DOCR	Duration of complete response
DOR	Duration of response
EBV	Epstein–Barr virus
ECOG	Eastern Cooperative Oncology Group
EFS	Event-free survival
FDA	Food and Drug Administration
FL	Follicular lymphoma
HCT	Hematopoietic cell transplantation
HCT-CI	Hematopoietic cell transplantation Comorbidity Index
HGBL	High-grade B-cell lymphoma
ICANS	Immune effector cell-associated neurotoxicity syndrome
IQR	Interquartile range
LBCL	Large B-cell lymphoma
LDH	Lactate dehydrogenase
MDS	Myelodysplasia
NE	Not evaluable
NMDP	National Marrow Donor Program
NOS	Not otherwise specified
NRM	Non-relapse mortality
ORR	Overall response rate
OS	Overall survival
PD	Progressive disease
PFS	Progression free survival
PMBCL	Primary mediastinal B-cell lymphoma
PR	Partial response
PTLD	Post-transplant lymphoproliferative disorder
REL/PD	Relapse or progressive disease
REMS	Risk Evaluation and Mitigation Strategy
R/R LBCL	Relapsed or refractory large B-cell lymphoma
SAS	Statistical Analysis System
SD	Standard deviation
TTCR	Time to complete response
TTOR	Time to overall response
WBC	White blood cell

## Appendix B

**Table A1:** Reasons for ZUMA-1 ineligibility.

Reason	*N* (%), *N* = 39
Other B-cell lymphoma	1 (3)
ECOG > 1	7 (18)
Organ impairment	28 (72)
Renal (moderate/severe), at the time of infusion or prior renal transplant	2 (5)
Hepatic (moderate/severe), any history or at the time of infusion	1 (3)
Pulmonary (moderate), at the time of infusion	12 (31)
Pulmonary (severe), at the time of infusion	4 (10)
Cardiac, any history	14 (36)
Cerebrovascular disease, any history	3 (8)
Heart valve disease	1 (3)
Infection	4 (10)
Autoimmune disease	4 (10)
CNS involvement	3 (8)

Abbreviations: CNS, central nervous system; ECOG, Eastern Cooperative Oncology Group.

**Table A2:** Primary cause of death.

Cause ^a^	*N* (%)
Primary disease	42 (37)
Organ failure ^b^	3 (3)
Malignancy ^c^	2 (2)

^a^ % of all patients. Number of patients who died during follow-up (*n* = 47). ^b^ Cases of organ failure were reported as two pulmonary failures and one cardiac failure, occurring at 4.2 months, 7.9 months, and 0.3 months, respectively. ^c^ One patient reported onset of MDS on Day 142, died on Day 435 without REL/PD of primary disease. One patient reported onset of AML on Day 160, died on Day 264 without REL/PD of primary disease. There were no malignancies of T cell origin.
