# Supplementary material for: Real-World Outcomes of Axicabtagene Ciloleucel for Treatment of Relapsed or Refractory Large B-Cell Lymphoma in Canada
Source: Curr Oncol. 2026 Jan 31;33(2):85. doi: 10.3390/curroncol33020085 (PMC12939802; doi:10.3390/curroncol33020085)
Supplement: Supplementary file 1 [file curroncol-33-00085-s001.zip › curroncol-4078902-supplementary.pdf]

Supplemental Methods

Data reporting requirements: The Full Analysis Set comprises all patients who meet the eligibility criteria and who were reported on Form 4000 (Cellular Therapy Essential Data Pre-Infusion Form), Form 2402 (Disease Classification), Form 2018 (Hodgkin and Non-Hodgkin Lymphoma Pre-Infusion), at least the first Form 4100 (Cellular Therapy Essential Data Follow-up Form) at 100 days and at least the first Form 2118 (Hodgkin and Non-Hodgkin Lymphoma Post-Infusion Data) at 100 days.

Supplemental Tables

Table S1: Efficacy outcomes by subgroups.

	ORR (%, 95% CI)	CR (%, 95% CI)	Time to Response (6m) (est %, 95% CI)	DOR (6m) (est %, 95% CI)	DOR (12m) (est %, 95% CI)	PFS (6m) (est %, 95% CI)	OS (6m) (est %, 95% CI)	OS (12m) (est %, 95% CI)	REL/PD (6m) (est %, 95% CI)	REL/PD (12m) (est %, 95% CI)	Non-relapse mortality (6m) (est %, 95% CI)	Non-relapse mortality (12m) (est %, 95% CI)
By HCT-CI prior to infusion												
0 (n = 48)	75 (60-86)	60 (45-74)	73 (57-84)	65 (45-79)	58 (37-75)	58 (43-71)	79 (64-88)	60 (43-73)	40 (26-53)	48 (32-62)	2 (< 1-10)	2 (< 1-10)
1 – 2 (n = 37)	81 (65-92)	57 (39-73)	80 (61-90)	68 (47-82)	54 (31-73)	59 (42-73)	75 (58-86)	64 (45-77)	38 (22-53)	44 (27-59)	3 (< 1-12)	3 (< 1-12)
3+ (n = 29)	76 (56-90)	59 (39-76)	NE (NE-NE)	62 (38-79)	51 (28-70)	58 (39-74)	76 (55-88)	52 (31-69)	42 (23-59)	49 (30-66)	0 (NE-NE)	8 (1-23)
By age at infusion												
< 65 (n = 69)	75 (64-85)	58 (45-70)	74 (61-83)	62 (47-75)	50 (33-65)	57 (44-67)	74 (61-83)	55 (42-66)	42 (30-53)	49 (36-60)	1 (< 1-7)	5 (1-12)
≥ 65 (n = 45)	80 (65-90)	60 (44-74)	79 (62-89)	68 (49-82)	60 (36-77)	62 (46-74)	82 (67-90)	65 (48-78)	36 (22-50)	44 (29-58)	2 (< 1-10)	2 (< 1-10)
< 75 (n = 102)	77 (68-85)	59 (49-68)	76 (66-84)	67 (54-76)	54 (40-67)	59 (49-68)	76 (67-83)	59 (48-68)	39 (30-49)	45 (35-55)	2 (< 1-6)	4 (1-10)
≥ 75 (n = 12)	75 (43-95)	58 (28-85)	75 (33-93)	49 (13-78)	49 (13-78)	57 (25-80)	83 (46-95)	62 (27-84)	43 (14-69)	66 (24-88)	0 (NE-NE)	0 (NE-NE)
ECOG PS prior to infusion												
0 or 1 (n=107)	79 (70-86)	62 (52-71)	77 (68-85)	68 (57-78)	57 (42-69)	61 (51-69)	52 (42-61)	78 (69-85)	60 (50-69)	38 (28-47)	44 (34-54)	2 (< 1-6)
≥ 2 (n=7)	57 (18-90)	14 (< 1-58)	57 (12-86)	0 (NE-NE)	0 (NE-NE)	29 (4-61)	14 (< 1-46)	57 (17-84)	43 (10-73)	71 (18-94)	86 (13-99)	0 (NE-NE)
By prior auto-HCT												
No (n=79)	75 (64-84)	57 (45-68)	74 (62-83)	64 (49-75)	56 (41-69)	54 (43-64)	47 (35-57)	74 (63-83)	58 (45-68)	43 (32-54)	49 (37-60)	3 (< 1-8)
Yes (n=34)	82 (65-93)	62 (44-78)	81 (60-91)	71 (48-85)	57 (32-76)	68 (49-81)	57 (39-72)	82 (64-92)	62 (42-76)	32 (17-48)	39 (23-56)	0 (NE-NE)
By time from initial diagnosis to infusion												
< 12 months (n=46)	76 (61-87)	52 (37-67)	75 (59-86)	60 (41-74)	46 (26-64)	52 (37-65)	44 (30-58)	67 (52-79)	54 (38-68)	46 (31-59)	51 (35-64)	2 (< 1-10)
≥ 12 months (n=68)	78 (66-87)	63 (51-75)	77 (64-86)	68 (53-80)	60 (41-74)	63 (50-73)	53 (40-64)	83 (72-90)	62 (49-73)	36 (24-47)	44 (32-56)	1 (< 1-7)
By number of lines of prior therapy												
1 – 2 (n = 47)	77 (62-88)	60 (44-74)	76 (60-86)	74 (55-85)	58 (36-75)	64 (48-76)	76 (61-86)	63 (46-75)	34 (21-48)	42 (27-56)	2 (< 1-10)	2 (< 1-10)
3+ (n = 55)	75 (61-85)	56 (42-70)	74 (60-84)	58 (41-72)	51 (31-68)	54 (40-67)	76 (62-85)	59 (44-71)	44 (30-56)	52 (38-65)	2 (< 1-9)	4 (< 1-12)

Abbreviations: CI, confidence interval; CR, complete response; DOR, duration of response; ECOG PS, Eastern Cooperative Oncology Group Performance Status; HCT-CI, hematopoietic cell transplantation-specific comorbidity index; NE, not evaluable; ORR, overall response rate; OS, overall survival; PFS, progression-free survival; REL/PD, relapse or progressive disease.

Table S2: Safety outcomes by subgroups.

	Any grade CRS (%, 95% CI)	Grade ≥ 3 CRS[b] (%, 95% CI)	Any grade ICANS (%, 95% CI)	Grade ≥ 3 ICANS[b] (%, 95% CI)	No. treated for CRS &/or ICANS (n/N)	Prolonged cytopenia[a] (n,%)	Clinically significant infection (n,%)
By HCT-CI prior to infusion							
0 (n = 48)	83 (69-93)	5 (< 1-16)	49 (33-65)	19 (8-33)	39/48	8 (17)	24 (50)
1 – 2 (n = 37)	67 (49-81)	3 (< 1-15)	29 (15-47)	6 (< 1-20)	25/37	6 (16)	15 (41)
3+ (n = 29)	81 (62-94)	0 (0-13)	31 (15-51)	3 (< 1-18)	23/29	5 (17)	14 (48)
By age at infusion							
< 65 (n = 69)	74 (62-84)	2 (< 1-9)	33 (22-45)	9 (3-18)	51/69	12 (17)	31 (45)
≥ 65 (n = 45)	81 (67-92)	5 (< 1-16)	46 (30-63)	13 (4-27)	36/45	7 (16)	22 (49)
< 75 (n = 102)	77 (67-85)	2 (< 1-7)	35 (26-46)	10 (5-18)	77/102	17 (17)	47 (46)
≥ 75 (n = 12)	82 (48-98)	9 (< 1-41)	60 (26-88)	10 (< 1-45)	10/12	2 (17)	6 (50)
ECOG PS prior to infusion							
0 or 1 (n = 107)	79 (69-86)	3 (< 1-9)	37 (28-48)	10 (5-18)	83/107	17 (16)	50 (47)
≥ 2 (n = 7)	50 (12-88)	0 (0-46)	43 (10-82)	14 (< 1-58)	4/7	2 (29)	3 (43)
By prior auto-HCT							
No (n = 79)	82 (72-90)	4 (< 1-11)	39 (28-51)	8 (3-17)	64/79	11 (14)	37 (47)
Yes (n = 34)	63 (44-80)	0 (0-12)	33 (18-52)	15 (5-32)	22/34	8 (24)	15 (44)
By time from initial diagnosis to infusion							
< 12 months (n = 46)	76 (61-88)	5 (< 1-16)	32 (18-48)	5 (< 1-17)	36/46	9 (20)	22 (48)
≥ 12 months (n = 68)	78 (66-87)	2 (< 1-9)	42 (29-54)	14 (7-25)	51/68	10 (15)	31 (46)
By # of lines of prior therapy							
1 – 2 (n = 47)	77 (62-89)	0 (0-8)	44 (28-60)	12 (4-26)	35/47	7 (15)	22 (47)
3+ (n = 55)	90 (78-97)	6 (1-17)	40 (26-54)	9 (3-21)	49/55	9 (17)	24 (44)

[a]Among patients who survived Day 30; [b]Missing were excluded. Abbreviations: CI, confidence interval; CRS, cytokine release syndrome; ECOG PS, Eastern Cooperative Oncology Group Performance Status; ICANS, immune effector cell-associated neurotoxicity syndrome; HCT-CI, hematopoietic cell transplantation-specific comorbidity index.
